# Correction: Antibacterial activity of a dual peptide targeting the *Escherichia coli* sliding clamp and the ribosome

**DOI:** 10.1039/d1cb90020j

**Published:** 2021-07-06

**Authors:** Christophe André, Florian Veillard, Philippe Wolff, Anne-Marie Lobstein, Guillaume Compain, Clément Monsarrat, Jean-Marc Reichhart, Dominique Y. Burnouf, Gilles Guichard, Jérôme E. Wagner

**Affiliations:** Univ. Bordeaux, CNRS, Bordeaux INP, CBMN, UMR 5248, Institut Européen de Chimie et Biologie 2 rue Robert Escarpit F-33607 Pessac France g.guichard@iecb.u-bordeaux.fr; Insect Models of Innate Immunity, UPR 9022-CNRS, Institut de Biologie Moléculaire et Cellulaire 67000 Strasbourg France; CNRS, Architecture et Réactivité de l’ARN, UPR 9002, Institut de Biologie Moléculaire et Cellulaire 67000 Strasbourg France d.burnouf@ibmc-cnrs.unistra.fr; Université de Strasbourg, CNRS, Biotechnologie et Signalisation Cellulaire, UMR 7242, Ecole Supérieure de Biotechnologie de Strasbourg 67400 Illkirch France jerome.wagner@unistra.fr

## Abstract

Correction for ‘Antibacterial activity of a dual peptide targeting the *Escherichia coli* sliding clamp and the ribosome’ by Christophe André *et al.*, *RSC Chem. Biol.*, 2020, **1**, 137–147, DOI: 10.1039/D0CB00060D.

Camille Noûs, listed as the 8th author on the original paper, is a figure representing a collective and does not meet the author guidelines of the journal. Camille Noûs has therefore been removed as an author on the paper, along with the associated affiliation. The corrected list of authors and affiliations for this paper is shown above.

The Royal Society of Chemistry apologises for these errors and any consequent inconvenience to authors and readers.

## Supplementary Material

